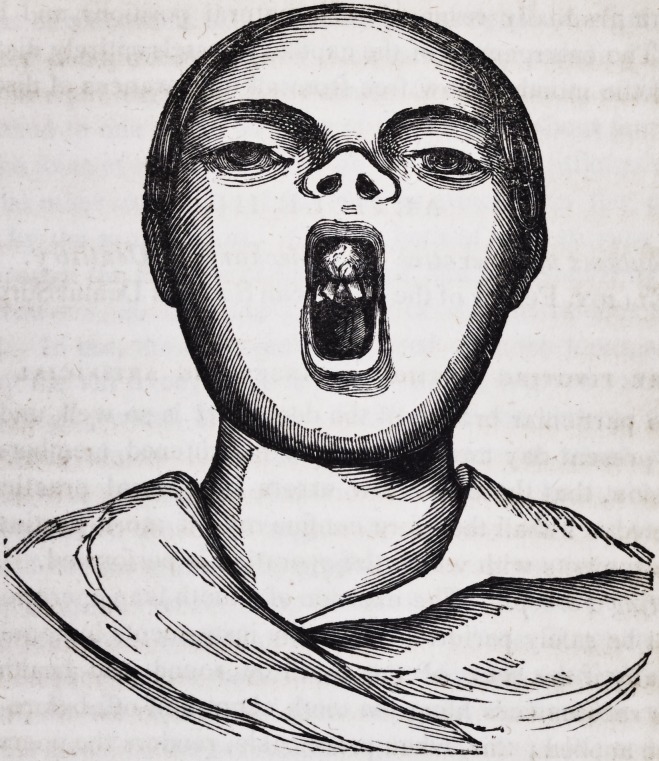# Case of Aneurism by Anastomosis of the Superior Maxillare

**Published:** 1844-03

**Authors:** S. P. Hullihen

**Affiliations:** Wheeling, Va.


### ARTICLE II.

*Case of Aneurism by Anastomosis of the Superior Maxillare.* By S. P. HULLIHEN, D. D. S., Wheeling, Va.

THE following case of aneurism by anastomosis, is reported more for the singularity of its situation than for any thing connected with its history, treatment, or cure.

In the autumn of 1841, Mrs. Stoneman, of this city, aged 22, pregnant with her first child, and having *nævi materni*, or mother's mark, on the left side of the nose and upper lip, showed me two small and very red protuberances or tumors of the gum, nearly of the same size. The larger was situated in the gum on the anterior surface of the alveolar process of the superior maxillare, and between the fangs of the central incisors. The smaller occupied the same situation on the posterior surface of the same process. A small cord-like enlargement ran from one tumor to the other, forcing the central incisors slightly apart. This constituted all the peculiarities of the case at this time.

The history of the case is as follows: during the first two months of her pregnancy, she had frequent and spontaneous bleedings from between the central incisors; after this, the small red tumors suddenly made their appearance, at which she became alarmed, and sought advice; but not being willing to submit to the treatment recommended, nothing more was heard from her until August, 1843. She was then five months pregnant with her second child, and had suffered much from frequent bleedings ever since the first appearance of the disease. The size of the tumors, *or rather tumor,* remained nearly stationary after the birth of her first child, until her second pregnancy occurred, and then it began gradually to enlarge, and was now the size of a filbert on the anterior surface, and about half this size on the posterior surface of the alveolar process. The cord-like enlargement or portion of tumor that passed between the central incisors, had so increased in size, as to force the teeth one-third of an inch apart, causing them and the lateral incisors to become very loose, sore, and painful. The tumor was at this time of a dark purple color, very soft, and pulsated as distinctly as the artery at the wrist. The capillary vessels on the upper lip were enormously enlarged, some distance on each side of the median line. The appearance of the case is well represented by the annexed cut.

As the patient was now suffering considerable pain, and the bleedings from the tumor were so frequent and so profuse as to affect her health very seriously, an operation was determined upon, and performed in the following manner:

A strong pin was pushed deeply through a portion of the gum at the upper edge of the tumor on the anterior surface of the alveolar process, and parallel with the jaw. Another pin, after being bent to suit the arch of the jaw, was inserted in the same way at the upper edge of the tumor on the inside of the mouth. A strong ligature was then thrown around the ends of the pins, and carefully pushed down between the tumor and the teeth, drawn very tight, and secured by a knot. In this way the whole tumor was embraced in one ligature, and in a manner that prevented the ligature from slipping or becoming displaced. On the second day the ligature was tightened, and also on the fourth and fifth. On the sixth day the tumor came away, and not a vestige of the disease remained. The parts healed up rapidly; the teeth gradually resumed their natural positions and became firm. The enlargement of the capillary vessels entirely disappeared, and the mouth is now free from all appearances of disease.

## Figures and Tables

**Figure f1:**